# Medicinal Plants Used for the Treatment of Erectile Dysfunction in Ethiopia: A Systematic Review

**DOI:** 10.1155/2021/6656406

**Published:** 2021-06-07

**Authors:** Demoze Asmerom, Tesfay Haile Kalay, Tsgabu Yohannes Araya, Desilu Mahari Desta, Dawit Zewdu Wondafrash, Gebrehiwot Gebremedhin Tafere

**Affiliations:** ^1^Department of Medicinal Chemistry, School of Pharmacy, College of Health Sciences, Mekelle University, P.O. Box 1871, Mekelle, Ethiopia; ^2^Department of Pharmacognosy, School of Pharmacy, College of Health Sciences, Mekelle University, P.O. Box 1871, Mekelle, Ethiopia; ^3^Clinical Pharmacy Unit and Research Team, School of Pharmacy, College of Health Sciences, Mekelle University, P.O. Box 1871, Mekelle, Ethiopia; ^4^Department of Pharmacology and Toxicology, School of Pharmacy, College of Health Sciences, Mekelle University, P.O. Box 1871, Mekelle, Ethiopia

## Abstract

**Background:**

Erectile dysfunction has remained as one of the major global health issues. Since the discovery of phosphodiesterase type 5 inhibitors, a significant portion of the patients has solved the issue of erectile dysfunction. However, the wide distribution of phosphodiesterase type 5 enzymes at various sites of the body led phosphodiesterase type 5 inhibitors to cause various unnecessary outcomes. Hence, it is vital to look for and find optional agents that could solve these limitations. The people of Ethiopia depend heavily on medicinal plants to ease their ailments, including erectile dysfunction. *Aim of the study*. The current study was carried out to systematically review the traditional medicinal plants used for the management of erectile dysfunction in Ethiopia.

**Method:**

A systematic and manual search was conducted to retrieve relevant articles published from 2000 to August 2020. Electronic databases of PubMed (Medline), Google Scholar, and grey literature were employed to access the studies. Accordingly, fifty-four published articles and thesis papers were finally included in this study.

**Result:**

Seventy plant species have been reported for the management of erectile dysfunction in Ethiopia. The commonly recorded family was Fabaceae, followed by Asteraceae, Malvaceae, Convolvulaceae, and Solanaceae. The plant species that represented the highest number of citations were *Asparagus africanus*, succeeded by *Ricinus communis* and *Carissa spinarum*. The commonest plant part used was roots. Majority of the medicinal plants were administered orally. The growth forms of the reported species were primarily herbs followed by shrubs.

**Conclusion:**

The present review compiled medicinal plants utilized by the Ethiopian community to manage erectile dysfunction. The findings will serve as a reference for the selection of plants for further pharmacological, toxicological, and phytochemical investigations in developing new plant-based drugs used for the treatment of erectile dysfunction.

## 1. Introduction

Erectile dysfunction (ED) (also called impotence) is the inability to achieve or maintain an erection sufficient for satisfactory sexual performance [1]. It has remained one of the major global health issues which is usually attributed to age, diabetes mellitus, smoking, cardiovascular diseases, kidney disease, previous operations, psychological factors, and drugs [2, 3]. Previously, about 52% of ED in men was seen in the age range of 40 to 70 years [3]. However, recent studies reported that ED is becoming highly prevalent even under the age of 40 [4]. In Africa, around 71.45% of people with diabetes developed ED [5]. In Ethiopia, about 60.4% of diabetic patients were reported with varying degrees of ED and the majority of the patients did not receive any medications [6]. More terribly, if this is not halted as early as possible, the number of ED cases globally is predicted to be 322 million by 2025 [7].

Erectile dysfunction can be managed nonpharmacologically via controlling plasma glucose levels and lipid profiles, avoiding smoking and alcohol drinking, psychological therapy, physical exercising, and external devices [8, 9]. Pharmacologically, it can be treated with different drugs including phosphodiesterase type 5 inhibitors (PDE5-Is), such as sildenafil, vardenafil, and tadalafil; apomorphine; and synthetic prostaglandin E1 (alprostadil), phentolamine, and papaverine [8, 10]. Of those, PDE5-Is are the most commonly suggested and used first-line treatment options in the world. However, the wide distribution of phosphodiesterase type 5 gene at various sites of the body led PDE5-Is to cause various adverse effects such as headache, myalgia, facial flushing, heartburn, nasal congestion, and vision-related problems. Moreover, disease conditions affecting the upstream nitric oxide pathways have been found with loss of efficacy [10]. Hence, it is vital to look for and find optional agents that could solve these limitations.

Since immemorial times, plants have been used as medicines to treat a myriad of human afflictions. This is because plants are a bank of bioactive compounds responsible for mitigating various disease conditions [11]. The people of Ethiopia depend heavily on medicinal plants to ease their ailments [12]. In Ethiopia, there are also more traditional healers than modern physicians [13]. Furthermore, traditional medicinal plants are considered as accessible, affordable, and acceptable in the community [14]. Around 6500 plant species are reported in the Ethiopian flora; of those, approximately 12% are endemic. In those Ethiopian floras, about 1000 plant species are identified as medicinal plants. However, the majority of the plant species are not yet identified [15]. This highlights that screening of the Ethiopian plants might grant various novel structures that might be unlikely to be discovered from other sources; ultimately, they may serve as lead compounds to fight various ailments including ED. Hence, documenting, compiling, and then assessing the effect of traditionally claimed plant species are worthwhile to come up with novel plant-based therapies.

## 2. Aim of the Study

The current study was carried out to systematically compile and document the traditional medicinal plants used for the management of ED or impotence in Ethiopia. The central thesis of this paper is therefore to encourage researchers to scientifically confirm the effect of medicinal plants against the global issue of ED.

## 3. Methods

**Figure 1:**
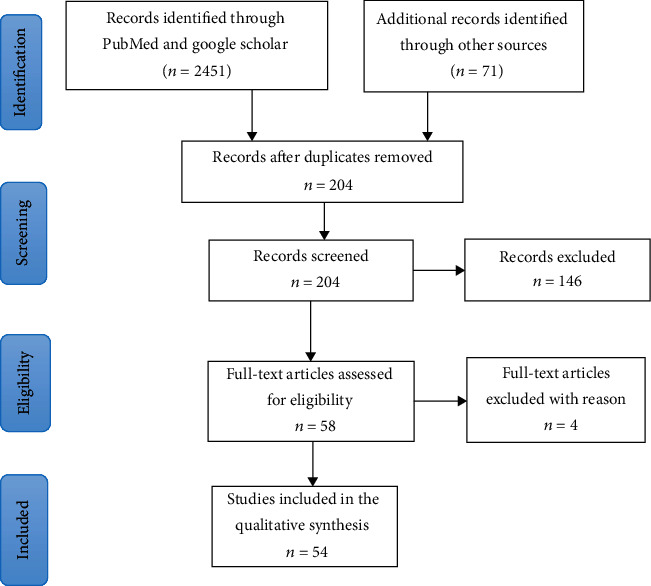
Study flow diagram.

This review was carried out following the recommendations stated in the Preferred Reporting Items for Systematic Reviews and Meta-Analyses (PRISMA) statement [16]. The search strategy flow chart is presented in Figure 1.

### 3.1. Search Strategy

A web-based systematic research literature search strategy was conducted through various electronic databases including PubMed (Medline), Google Scholar, and grey literature to access the relevant studies. The following search terms and combinations were used to collect relevant results: erectile dysfunction, impotence, traditional medicine, medicinal plants, ethnomedicine, ethnobotany, ethnopharmacology, indigenous, folk medicine, home remedy, herbal medicine, and Ethiopia.

### 3.2. Study Selection

#### 3.2.1. Inclusion Criteria

Original published articles and thesis dissertations conducted over the period from 2000 to August 2020 were only searched. The studies written in the English language were only searched. Finally, studies with Ethiopian traditional medicinal plants exclusively utilized for the treatment of ED/impotency in humans were selected.

#### 3.2.2. Exclusion Criteria

Articles pertaining outside Ethiopia, pharmacological studies, ethnoveterinary studies, and reviewed papers were excluded. Besides, the studies failed to mention the scientific name of the plant and the plant parts used were excluded from this study.

### 3.3. Data Retrieval

Studies that have possessed the required information are extracted. The required information was the family name, scientific name, local name (if available), habitat, parts used, method(s) of preparation (if available), and mode of administration. In case of missed information in some studies, especially the habitat of the plants, family name, and misspelled scientific name, information was retrieved from the Global Plants Journal of Storage (JSTOR) database [17].

### 3.4. Data Analysis

Microsoft Excel 2016 was employed to analyze the frequency distribution of families, plant parts, routes of administration, and habits. Besides, the distribution in regions where the medicinal plants were reported was analyzed. The results were depicted in charts and tables.

## 4. Results and Discussion

### 4.1. Distribution of Medicinal Plants

**Figure 2:**
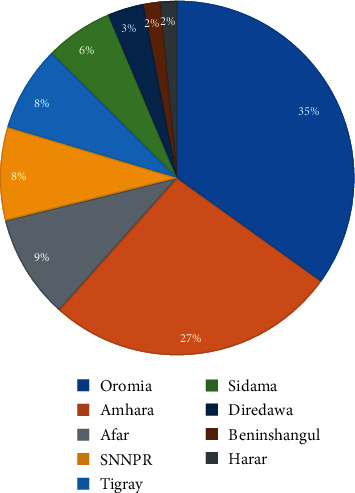
Distribution of medicinal plants across regions of Ethiopia.

The regions of Ethiopia that showed the highest ethnobotanical records were Oromia (35%) and Amhara (27%) that constituted about two-thirds (62%) of the total ethnobotanical records against ED (Figure 2). Several medicinal plants have been found in the Oromia region, according to most studies. This may be because, in addition to having a large number of traditional healers, those regions are also Ethiopia's most populous [18]. However, studies on the prevalence of ED in different regions of Ethiopia are limited.

### 4.2. Diversity of Medicinal Plants

**Figure 3:**
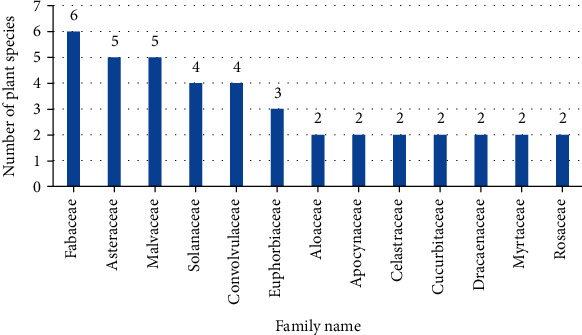
Diversity of medicinal plants used for the management of ED, for families with more than two species.

As shown in Table 1, the current review reported 70 Ethiopian plant species that have traditionally been used to treat ED. The top recorded families were Fabaceae (6 species), Asteraceae (5 species), Malvaceae (5 species), Convolvulaceae (4 species), Solanaceae (4 species), and Euphorbiaceae (3 species) (Figure 3). Alike this study, Semenya and Potgieter [19] reported that Fabaceae and Asteraceae were among the commonly used families for ED. Ajao et al. [20] also stated that medicinal plants under Fabaceae were the top species used for the management of ED in Sub-Saharan Africa. Moreover, the root of *Eriosema kraussianum* N. E. Br., Fabaceae, displayed a promising effect for ED in experimental rat models [21]. According to a recent study in Ethiopia, plants in the Fabaceae family are the most commonly used traditional medicinal plants [18]. As a result, these studies highlight the screening of plant species belonging to the Fabaceae family that could be important candidates to bring lead compounds to be used for future optional agents.

### 4.3. Frequently Used Medicinal Plants

The plant species that represented the highest number of citations were *Asparagus africanus* Lam. (8 citations), *Ricinus communis* L. (6 citations), and *Carissa spinarum* L. (4 citations), as well as *Ferula communis* L., *Aloe macrocarpa* Tod., and *Tragia brevipes* Pax with three citations each. Congruent to the present study, the people of Nigeria also traditionally use the root of *Asparagus africanus* Lam. for the management of ED [75]. The usage of this plant for the treatment of ED might be due to the presence of saponins [76], because plant species with saponins as their major constituent displayed significant promotion of erection [77]. The second most cited plant species is *Ricinus communis* L. (also known as castor bean). Recent *in vivo* studies of *Ricinus communis* L. have confirmed that it increases serum testosterone levels and multiple majors of sexual activity, supporting the current conventional claim [78]. The third cited plant, *Carissa spinarum* L., alike the Ethiopian people, the people of South and Central Benin use its roots for the treatment of sexual weakness. As a result, scientific evaluation of these claimed species is needed in order to uncover important leads in the fight against ED.

Plant species like *Syzygium aromaticum* L., *Zingiber officinale* Roscoe, and *Gloriosa superba* L are traditionally claimed in Ethiopia; they scientifically displayed significant aphrodisiac effect. That is, 50% ethanolic extract of *Syzygium aromaticum* L., (oral; 100, 250, and 500 mg/kg to rats) improved libido and erection, intromission frequency, mounting behavior, and mating performance [79, 80]. Hexane extract of the flower bud of *Syzygium aromaticum* (L.) Merr. & Perry. (clove) (oral; 15 mg/kg to mice) raised delta (5) 3-beta and 17-beta-hydroxysteroid dehydrogenase (Δ^5^, 3 *β*-HSD, and 17 *β*-HSD) and serum levels of testosterone [81]. Aqueous extract of Zingiber officinale (oral; 600 mg/kg to male *Wistar* rats) was tested for its possible androgenic activity and increased testis relative weight, serum testosterone, testicular cholesterol, and epididymal *α*-glucosidase activity [82]. Aqueous, chloroform, and alcohol extracts of *Gloriosa superba* at the dose of 500 mg/kg body weight showed an aphrodisiac effect with an increase in sexual and orientation behavior. Its aphrodisiac effect could be due to the presence of steroids, saponins, and alkaloids [83]. Hence, these studies support the acclaimed use of these plant species as a treatment for sexual dysfunction in Ethiopia.

These days, in Ethiopia, the continuation of traditional plant remedies is highly threatened due to deforestation, overgrazing, environmental degradation, agricultural expansion, and the rise of the population [15]. This, in turn, jeopardizes the extinction of essential medicinal plants which may have stored indispensable compounds that are responsible for addressing the existing global health issues. Therefore, early detection of the pharmacological activities of the reported species against ED is strongly recommended.

### 4.4. Growth Forms of the Medicinal Plants

**Figure 4:**
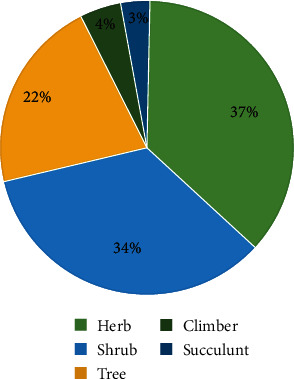
Growth forms of medicinal plants.

The growth forms of the reported species were herb (37%), shrub (34%), tree (22%), climber (4%), and succulent (3%) (Figure 4). This study is consistent with studies conducted by Worku [12] and Yirgu et al. [18] who reported that herbs were the most dominant plant growth forms as well as used as remedies in the Ethiopian traditional medicine. The highest use of herbaceous plants as compared to other growth forms could be due to their accessibility, the higher possibility of obtaining pharmacologically active compounds, and the sociocultural beliefs and practices of the healers in treating the ailment [84].

### 4.5. Plant Parts Used

**Figure 5:**
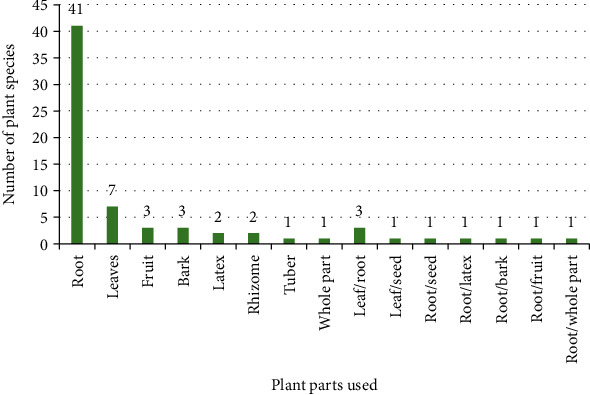
Plant parts used for ED.

The most common plant part used was root (41 species), followed by leaves (7 species), fruit (3 species), and bark (3 species) (Figure 5). Similarly, in another study, it was reported that the root was the predominant plant part used for the management of ED [85]. The people of South Africa, Limpopo province, also use roots as the most preferred medicinal plant part [19]. In contrast to this study, the people of Western Uganda use leaves as the commonest plant part for ED [86]. Irrespective of the dominancy, however, confirming the pharmacological activity of the claimed plant part is necessary, because most plant parts reside several bioactive principles.

### 4.6. Mode of Administration

**Figure 6:**
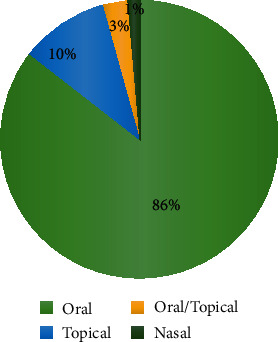
Mode of administration of the medicinal plant.

The most common route of administration of the medicinal plants was oral (86%), followed by topical (10%), oral/topical (3%), and nasal (1%) (Figure 6). In agreement with this study, Semenya and Potgieter [19] mentioned the oral route as the dominant route for ED. The commonly reported cosolvents were “*tella* (local drink)” (8 species), butter, honey (5 species), and coffee (4 species).

## 5. Conclusion

The present review compiles and documents for the first time seventy (70) medicinal plant species used for the management of ED in Ethiopia. Fabaceae was the dominant plant family used for the management of ED in Ethiopia. *Asparagus africanus* was the most repeatedly cited plant species against ED. Plant species like *Syzygium aromaticum* L., *Zingiber officinale* Roscoe, and *Gloriosa superba* L. are traditionally claimed in Ethiopia; they scientifically displayed significant aphrodisiac effect. This suggests the reported plant species could be a source of a new class of drugs against ED. Thus, the current findings may serve as references for the selection of plants for further pharmacological, toxicological, and phytochemical investigations in developing new plant-based drugs used for the treatment of ED.

## Figures and Tables

**Table 1:** Medicinal plants used for the treatment of erectile dysfunction in Ethiopia.

S. no..	Scientific name	Family name	Local name	Habit	PU	Method of preparation	ROA	References
1	*Acacia mellifera* Benth	Fabaceae	*Kontir grar* (Ha)	T	Root	Taken with the root and barks of *Amaranthus cruentus*	Oral	[22, 23]
2	*Acacia senegal* (L.) Wild	Fabaceae	Not mentioned	T	Root	Not mentioned	Oral, topical	[24]
3	*Achyranthes aspera* L.	Amaranthaceae	*Darguu* (Or)	H	Root	Not mentioned	Oral	[25]
4	*Adansonia didgitata* L.	Bombacaceae	*Dima* (Tg)	T	Root	Crush, mix with honey, and eat the mixture before break fast	Oral	[26]
5	*Aloe macrocarpa* Tod.	Aloaceae	*Ret/eret* (Am)	Su	Latex	The latex is mixed with butter and use it to stain the whole part of the penis and heat it with fire for continuous days	Topical	[27–29]
6	*Aloe megalacantha* Baker	Aloaceae	*Ere* (Tg)	Su	Latex/root	Smearing penis with exudate	Topical	[30, 31]
7	*Asparagus africanus* Lam.	Asparagaceae	*Kasta ansti* (Tg), Sariti, *Yeset qest* (Am)	S	Root/leaf	(i) Roots are pound into powder, mixed with meat soup and vegetable, and then taken every evening for a month (ii) Leaf powder is mixed with butter and drank for 3 days before sexual intercourse (iii) The root together with roots of *Premna schimperi* and *Olea europaea* are pound and given to the victim with one cup of “*tella*” (local alcohol) 2–3 hrs before sexual works	Oral	[25, 28, 31–36]
8	*Cadaba farinosa* Forssk.	Capparidaceae	Not mentioned	S	Root	Not mentioned	Oral	[24]
9	*Calpurnia aurea* (Aiti) Benth.	Fabaceae	*Ceekataa* (Sd)	S	Root/seeds	(i) Root tip is chewed and the juice is drank with an alcoholic drink (ii) Crushed, powdered, mixed with water, fermented overnight, and drank	Oral	[37, 38]
10	*Capparis tomentosa* Lam.	Capparaceae	*Gimero* (Am)	S	Root	Powder paste with butter applied on the penis (glans)	Topical	[39]
11	*Capsicum annuum* L.	Solanaceae	*Mixxamixxoae* (Ko)	H	Fruit	Fruit is eaten	Oral	[36]
12	*Carissa spinarum* L.	Apocynaceae	*Hagamsa* (Or)	S	Root/bark	(i) Fresh root is pounded and mixed with “*tella*” (ii) Its bark and the bark of *Pavetta abyssinica* are mixed and then powdered, cooked, and taken orally	Oral	[25, 40–42]
13	*Carthamus lanatus* L.	Asteraceae	Not mentioned	H	Leaf	It is crushed and pounded with the whole parts of *Trajia cinerea* and the root of *Hibiscus eriospermus* and then stirred in a local beer and drank in one cup of coffee until recovery	Oral	[43]
14	*Catha edulis* (Vahl) Forssk.	Celastraceae	*Chat* (Am)	S	Leaf	Decoction	Not mentioned	[25, 39]
15	*Caylusea abyssinica* (Fresen.) Fisch. & Mey.	Resedaceae	*Reenci* (Or)	H	Root	Drinking the powdered root with water and/or using it for toothbrush daily	Oral	[32, 35]
16	*Chlorophytum laxum* R. Br	Liliaceae	*Munna* (Sh)	H	Tuber	Tuber is eaten cooked	Oral	[44]
17	*Clausena anisata* (Willd.) Benth	Rutaceae	*Ulumayii* (Or)	S	Root	Not mentioned	Oral	[25]
18	*Convolvulus arvensis* L.	Convolvulaceae	*Este filastot* (Am)	H	Root	Crush and powder then drink with GIN (*areki*)	Oral	[28]
19	*Crotalaria spinosa* Hochst. ex Benth	Fabaceae	*Chifrig* (Tg)	H	Root	Crushing, mixing and eat	Oral	[26]
20	*Drymaria cordata* (L). Schultes.	Caryophyllaceae	*Saydasajal* (Or)	H	Root	Cutting, with bulbs of *Zingiber officinale* and *Allium sativum* and then eating by spoon	Oral	[45]
21	*Euclea racemosa* Murr. subsp. *Schimperi* (A. DC.) F. White	Ebenaceae	*Kulio* (Tg)	S	Root	Crush, add to the chicken stew, and eat with injera (local meal) for 7 days before the meal	Oral	[26]
22	*Euphorbia tirucalli* L.	Euphorbiaceae	*Kenchib* (Tig)	T	Latex	The fresh latex is mixed with butter and used to stain the whole part of the penis and heated for about 5 minutes for 3 days	Topical	[46]
23	*Falkia canescens* C.H. Wright	Convolvulaceae	*Gura hantutaa* (Or)	H	Leaf	Crushed, mixed with butter, and eaten for 5 days	Oral	[47]
24	*Ferula communis* L.	Apiaceae	*Dog* (Am)	H	Root	Powderize the concoction then drinks with “*tella*”	Oral	[28, 48, 49]
25	*Ficus sur* Forssk.	Moraceae	*Harbuu* (Or)	T	Root	Not mentioned	Oral	[25]
26	*Garcinia buchananii* Baker	Clusiaceae	*Soloolsa* (Sd)	T	Bark	The bark is peeled carefully, boiled, cooled, and drunk	Oral	[50]
27	*Gloriosa superba* L.	Colchicaceae	*Yebab Mashila* (Am)	S	Root	The root powder is taken with “*tej*” for 3 days	Oral	[51]
28	*Gomphocarpus stenophyllus* Oliv.	Apocynaceae	*Chifirig* (Am)	S	Root	Maceration, taken orally once daily for seven days	Oral	[52]
29	*Grewia villosa* Willd.	Tiliaceae	Not mentioned	S	Root	Not mentioned	Oral, body wash	[24]
30	*Hibiscus eriospermus*	Malvaceae	Not mentioned	H	Root	It is the same method and ingredient used in *C. lanatus*	Oral	[43]
31	*Kalanchoe petitiana* A. Rich.	Crassulaceae	*Andahula* (Am)	H	Root	Milk decoction of the fresh pulverized roots and leaves	Oral	[53]
32	*Kleinia abyssinica* (A. Rich.) A. Berger	Asteraceae	*Abrasha* (Or)	H	Rhizome	Aphrodisiac fresh rhizome is eaten a few hours before sexual performance	Oral	[54]
33	*Lobelia gibberoa* Hemsl.	Lobeliaceae	*Jibara* (Am)	T	Root	Crush and then mix with coffee and drink	Oral	[48]
34	*Lagenaria siceraria* (Molina) Standl.	Cucurbitaceae	*Buqqee/Kil* (Or)	H	Root/fruit	The root and fruit are ground together and drank with the first boiled coffee	Oral	[55]
35	*Maytenus senegalensis* (Lam) Exell	Celastraceae	*Koba* (Am)	T	Bark	Dried stem bark powder cooked with hen meat is given orally	Oral	[56]
36	*Millettia ferruginea* (Hochst.) Bak.^∗^	Fabaceae	*Birbira* (Am)	T	Root	Not mentioned	Oral	[49]
37	*Nicotiana glauca* Grah.	Solanaceae	*Yeareb* Kitel (Am)	H	Leaf	Chewing very small pieces of leaf and swallowed	Oral	[57]
38	*Olea europaea* L. subsp. *cuspidata* Wall. ex G. Don	Oleaceae	*Ejersa* (Or)	T	Root	The root together with roots of *Aloe macrocarpa* and *Premna schimperi* pounded in water and given to the victim with “*tella*” before bed for a few days	Oral	[32, 35]
39	*Pavonia urens* Cav.	Malvaceae	*Ablalit* (Am)	S	Root	Root powder is taken with “*tella*” orally	Oral	[58]
40	*Periploca linearifolia* Quart.-Dill. and A. Rich.	Asclepiadaceae	*Tikur Areg* (Am)	Cl	Root	Dried or fresh root is chopped and tied on the waist	Topical	[59]
41	*Phoenix reclinata* Jacq.	Arecaceae	*Seniel* (Am)	T	Root	Not mentioned	Oral	[49]
42	*Plumbago zeylanica* L.	Plumbaginaceae	*Amira* (Am)	S	Leaf/root	Fresh leaf crushed and mixed with water	Oral	[60, 61]
43	*Prunus Africana* (Hook. f.) Kalkm.	Rosaceae	Not mentioned	T	Root	Fresh roots are crushed and soaked in water and then one cup is drunk	Oral	[62]
44	*Ricinus communis* L.	Euphorbiaceae	*Qobbo* (Or), *Gullo* (Am)	S	Leaf/seed	(i) Crushed leaves with coffee, tea, or milk are taken as a drink before copulation (ii) The dried seeds are pounded, mixed with a small quantity of latex from *Aloe* spp. and two coffee cups are drank before bedtime for two days	Oral	[63–68]
45	*Rosa abyssinica* Lindley	Rosaceae	*Gora* (Or)	S	Root	Not mentioned	Oral	[25]
46	*Sansevieria ehrenbergii* Schweinf. ex Baker	Dracaenaceae	*Wondiekacha* (Am)	H	Root	Not mentioned	Oral	[49]
47	*Sansevieria erythraeae* Mattei	Dracaenaceae	*Algeti/cheret* (Am)	H	Root	Root powder is taken with “*tef*” potage	Oral	[58]
48	*Seddera bagshawei* Rendle	Convolvulaceae	Not mentioned	S	Root	Not mentioned	Nasal	[24]
49	*Seddera hirsute* Dammer ex Hall. f.	Convolvulaceae	*Bikiltafri* (Af)	S	Whole/root	(i) The fresh whole plant is pounded, mixed with sugar and goat's milk, and drunk (ii) The root is chewed	Oral	[24, 69]
50	*Sida schimperiana* Hochst. ex A. Rich.	Malvaceae	*Chifrig* (Am)	S	Root	Roots are chewed and fluid swallowed	Oral	[70]
51	*Sida tenuicarpa* Vollesen	Malvaceae	*Chifrig* (Am)	S	Leaf	Boil leaf, mix with *N. Sativa* & leaf of *Withania* sp., *A. Sativum* & honey, and eat the mixture at a time of necessity	Oral	[71]
52	*Sida rhombifolia* L.	Malvaceae	*Gorgegit* (Am)	S	Root	Drink concoction with honey	Oral	[28]
53	*Solanum anguivi* Lam.	Solanaceae	*Zerch enbuay* (Am)	S	Root	Roots are chewed and fluid swallowed	Oral	[70]
54	*Stephania abyssinica* (Quart.-Dill. & A. Rich.) Walp.	Menispermaceae	*Harege-eyesus* (Sh)	Cl	Root	Not mentioned	Oral	[61]
55	*Syzygium aromaticum* L. Merr. & Perry.	Myrtaceae	*Kirunfud* (Am), *Qurunfudii* (Or)	T	Fruit	Dried fruit is crushed, mixed with goat milk, and boiled. Then, the decoction is drank	Oral	[66]
56	*Syzygium guineense* (Willd.) DC. *Subspafromontanum*	Myrtaceae	*Badessa* (Or)	T	Bark	Not mentioned	Oral	[25]
57	*Tamarindus indica* L.	Fabaceae	Not mentioned	T	Fruit	The fruit is chopped and taken orally with tea	Oral	[72]
58	*Tapinanthus globiferus* (A. Rich.) Tieghem	Loranthaceae	Not mentioned	H	Leaf	Not mentioned	Oral	[24]
59	*Thalictrum rhynchocarpum* Dill. & A. Rich.	Ranunculaceae	*Sire-bizu* (Am)	H	Root	Drink concoction with honey	Oral	[28]
60	*Tragia brevipes* Pax.	Euphorbiaceae	*Abelbalit* (Am)	H	Whole	(i) It is the same method and ingredient used in *C. lanatus* (ii) Chew and absorb the juice	Oral	[28, 43, 48]
61	*Tragia uncinate* M. Gilbert	Euphorbiaceae	*Amae* (Tg)	H	Root	Roots are ground and taken orally with local soup for a week	Oral	[31]
62	*Urtica simensis* Steudel.^∗^	Urticaceae	Doobii/Saammaa (Or)	H	Root	The root is chewed and the extract is swallowed	Oral	[73]
63	*Verbascum sinaiticum* Benth	Scrophulariaceae	*Ye Ahya joro* (Am), *Gurra Harree* (Or)	H	Root	Chopped leaf is rolled by a clean piece of cloth and tied around the male sex organ to erect it	Topical	[66]
64	*Verbena officinalis* L.	Verbenaceae	*Atuch* (Am)	H	Root	Drink concoction with honey	Oral	[28]
65	*Vernonia adonesis* Sch. Rip. ex Walp.	Asteraceae	*Pepa meta* (Gu), *Raskimir* (Am)	H	Root	Root is crushed and soaked in water (maceration) and one cup is taken	Oral	[56, 74]
66	*Vernonia amygdalina* Del.	Asteraceae	*Girawa* (Am)	S	Root	Drink the concoction with “*tella*”	Oral	[28]
67	*Vernonia myriantha* Hook. f.	Asteraceae	*Kotkoto* (Am)	S	Root	Drink the concoction with “*tella*”	Oral	[28]
68	*Withania somnifera* (L.) Dunal in DC	Solanaceae	*Giziewa* (Am)	S	Root	Drink the concoction with “*tella*”	Oral	[28]
69	*Zehneria scabra* (Linn. f.) Sond.	Cucurbitaceae	*Haregresa* (Am)	Cl	Leaf/root	Bathe in the infusion of leaf and root for 7 days	Topical	[53]
70	*Zingiber officinale* Roscoe	Zingiberaceae	*Jinjibelloae* (Ko)	H	Rhizome	Rhizomes are chewed and the exudates are swallowed	Oral	[36]

Habits—Cl: climber; H: herb; S: shrub; Su: succulent; T: tree; Language—Af: Afar; Am: Amharic; Gu: Gumuz; Ha: Hadiyigna; Ko: Koorete; Or: Oromiffa; Sd: Sidamigna; Sh: Shinasha; Tg: Tigrigna; ^∗^Endemic.

## Data Availability

The datasets used to support the findings of this study are available from the corresponding author upon request.

## Abbreviations

ED: Erectile dysfunction
PDE5-Is: Phosphodiesterase type 5 inhibitors.
